# Randomization in phase II trials: No exemption based on sample size

**DOI:** 10.1002/bcp.70167

**Published:** 2025-07-08

**Authors:** Theodor Framke, Gernot Beutel, Arnold Ganser, Armin Koch, Anika Großhennig

**Affiliations:** ^1^ Institute for Biostatistics Hannover Medical School Hannover Germany; ^2^ Department for Hematology, Hemostasis, Oncology and Stem Cell Transplantation Hannover Medical School Hannover Germany

**Keywords:** AML/MDS, clofarabine, drug development programme, evidentiary standards, fludarabine, phase 2, randomization

## Abstract

Conducting randomized clinical trials (RCTs) is challenging. For this reason, in earlier phases of drug development and rare diseases with limited sample sizes, there is a tendency to omit control groups and to replace them with historical information for perceived feasibility arguments. This has significant implications for interpretation of trial results. We present headline results from a trial in stem cell transplantation, where fludarabine and clofarabine were investigated for induction chemotherapy in high‐risk acute myeloid leukaemia or advanced myelodysplastic syndrome. The trial was initially designed as single‐arm trial (SAT) and is discussed here from the planning and assessment perspective. Changes in the treatment setting or patient selection may lead to ‘promising results’ in SATs, which are difficult to identify and may raise unwarranted hopes which cannot be confirmed in a subsequent randomized trial. RCTs should be used to achieve a straightforward interpretation of the results, even if the sample size is small. Interpreting SATs is only straightforward in very rare instances where the underlying treatment effect is large and the outcome in each individual patient surpasses all previous clinical experience.

**What is already known about this subject**

- The role of observational studies compared to studies with randomised treatment allocation has been discussed for decades.
- The shortcomings of single‐arm trials have been well documented in the past. However, in earlier phases of drug development or rare diseases, there is a tendency to omit control groups and to replace them with historical information for perceived feasibility arguments.

**What this study adds**

- This study reports headline‐results of a clinical trial in stem cell transplantation, where fludarabine and clofarabine were investigated for induction chemotherapy in high‐risk acute myeloid leukaemia or advanced myelodysplastic syndrome.
- We discuss the study from the perspective of what we would have concluded (if this study had been done as a single‐arm trial) and compare to the final outcome of the randomized clinical trial.
- We conclude that, a straightforward interpretation of single‐arm trials is only possible in very rare instances where the underlying treatment effect is extremely large and that even with small sample sizes, a randomized‐controlled trial is more informative.

## 1 INTRODUCTION

Randomized controlled trials (RCTs) are the ‘gold standard’ design. Drug development programmes based solely on single‐arm clinical trials (SATs) have been challenged for being prone to bias and inefficient. Nevertheless, there seems to be a paradigm shift from RCTs being essential to more drug approval decisions based on SATs that in instances have been retracted at a later point in time.^1^, ^2^

As a recent example, pembrolizumab, efficacious in many oncologic indications, was approved in 2017 for the treatment of patients with gastric or gastroesophageal junction adenocarcinoma. This decision was based on an open‐label, multicentre, non‐comparative, multi‐cohort trial in which 259 patients were included.^3^ Five years later this indication was withdrawn because a RCT demonstrated that pembrolizumab was not effective in patients with advanced gastric cancer.^4^

Here, we present the headline‐results of a study in haematology, which was initially planned as a single‐arm trial (SAT) and invite a discussion about the conclusions and implications if it had not been conducted as a RCT in the end.

## 2 THE CASE STUDY IN HAEMATOLOGY

ClAraC‐SCT enrolled patients with high‐risk acute myeloid leukaemia (AML) or advanced myelodysplastic syndrome (MDS) scheduled for stem cell transplantation (SCT). AML/MDS patient's prognosis is poor, and treatment options beyond stem‐cell transplantation are limited. The FLAMSA regimen, a triple‐combination including fludarabine, amsacrine and Ara‐C, is an established induction therapy for SCT.^5^, ^6^ However, especially in older patients and those with comorbidities, toxicity is a problem, motivating the continuous search for alternative approaches for induction chemotherapy. Subsequent retrospective data suggested that the combination of clofarabine and Ara‐C (ClAraC) could potentially provide a better toxicity profile and further improve the anti‐leukaemic efficacy, which is the key factor determining the success of the following SCT.^7^ Notably, treatment with ClAraC was already approved for use in paediatric patients with relapsed or refractory acute lymphoblastic leukaemia after at least two prior regimens.^8^, ^9^

During the planning stage, the conduct of a RCT was discussed, but as the available sample size was very small and given that only 30 patients could be recruited in a realistic timeframe, a small monocentric SAT was considered the preferred approach. Event‐free survival (EFS), defined as treatment failure, disease recurrence or death from any cause, was chosen as the primary endpoint. The evidence at the planning stage was based on the publication of results from two SATs in AML by Schmid et al.^6^, ^10^ They observed that the 2‐year leukaemia‐free survival (LFS) rates ranged from 37% and 40% and the 2‐year overall survival (OS) rates were in the range of 40% and 42%. Based on an exact binomial test, a two‐sided α of 5%, a power of 80% and a EFS rate of 66%, 30 patients are necessary to demonstrate a difference against an assumed historical null proportion of 39% (as the average of the two observed event rates).

**FIGURE 1:**
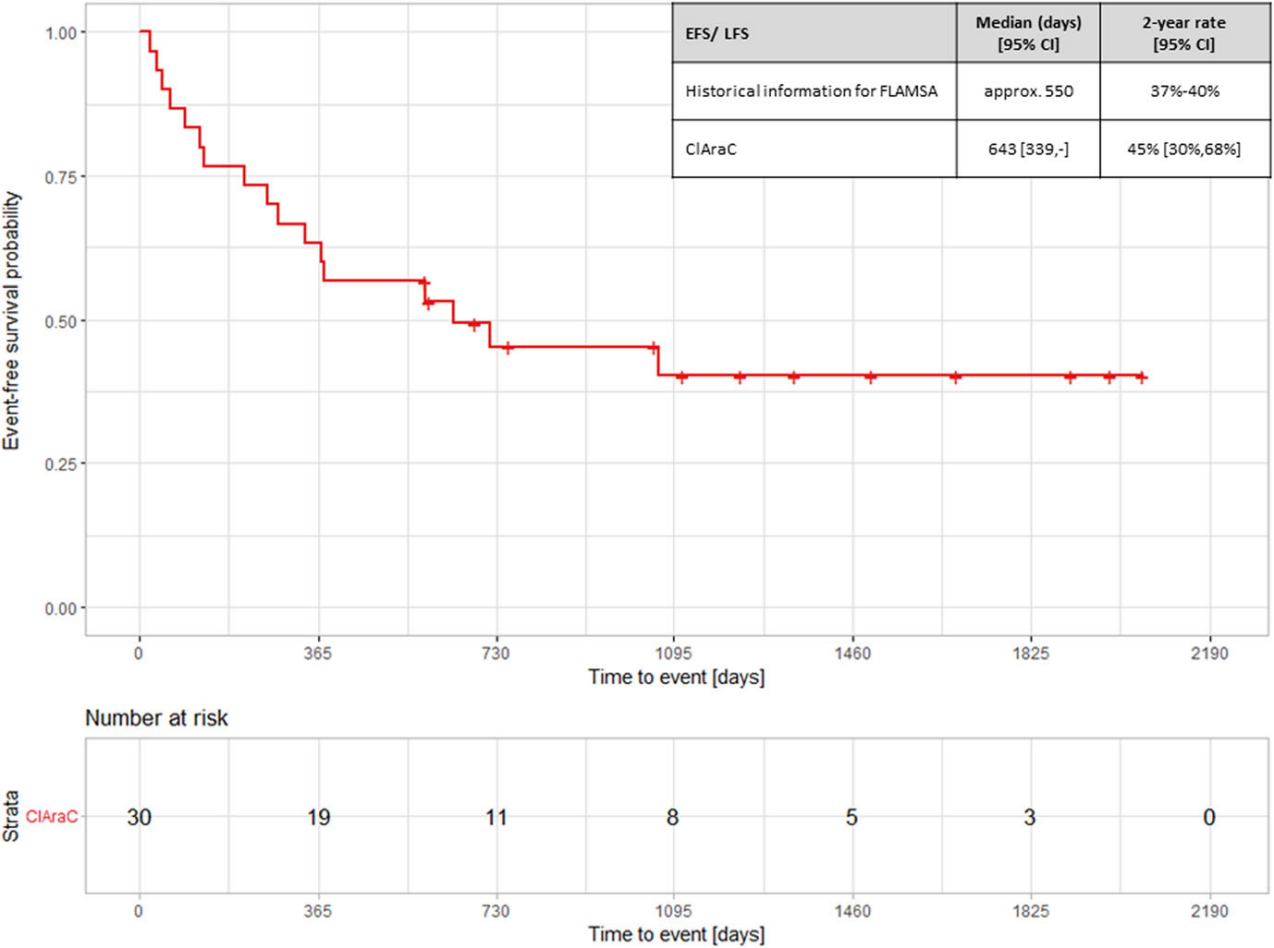
Results for event‐free survival of the ClAraC treatment group. Results of Kaplan–Meier analysis for the event‐free survival (EFS) of the ClAraC treatment group. Displayed are median survival and 2‐year event rates with 95% confidence intervals (CI) for EFS/LFS (leukaemia‐free survival) for ClAraC and information of historical control (FLAMSA).

The trial was conducted between 2011 and 2017 at Hannover Medical School. Figure 1 depicts the Kaplan–Meier curve for the EFS of the 30 patients treated with the ClAraC regimen. The observed EFS rate at 2 years was 45%, which is not extremely high but is numerically larger than the historically observed LFS and OS rates that we used at the planning stage.

In line with this, the OS rate was larger than the OS rate observed in the historical patients treated with FLAMSA, and no critical safety and toxicity issues were observed (see Appendix, Table S2). In summary, the results of the 30 patients treated with ClAraC regimen were not as good as hoped for and did not formally outperform the expectation based on the historical control data.

However, given the rarity of the condition and the existing unmet medical need, the implications of this study are obvious: EFS was better than the results of previously reported trials, leading to the question whether this was enough evidence to either use ClAraC for all patients on a regular basis or as an additional option for more frail patients. Or should this outcome in a very traditional approach now justify performing a second, larger and preferably RCT to compare FLAMSA and ClAraC directly? Obviously, this would mean accepting a delay of at least 6 years to ultimately answer the question whether ClAraC can be beneficially used in this indication. From this perspective, the decision problem after the conducted SAT is as difficult as before the trial, and the underlying research question remains unanswered.

In fact, this discussion was held at the trial design stage. Luckily, it was possible to gain the support of two further study sites, and at the end, even the recruitment of 60 patients was considered feasible. Instead of the monocentric SAT, a multicentre RCT was conducted, and 60 patients were randomly allocated to FLAMSA or ClAraC. The primary endpoint was EFS, defined as time until disease recurrence, treatment failure or death (due to any cause), whichever occurs first. Sample size planning was based on median survival times and performed using the log‐rank test.

In the end, treatment groups were well‐balanced for baseline variables (see Appendix, Table S1). Consort reporting guidelines and ICH E3 recommendations were applied for the final report of the study results.^11^, ^12^ The report is available from the DIMDI portal of the Federal Institute for Drugs and Medical Devices.^13^

**FIGURE 2:**
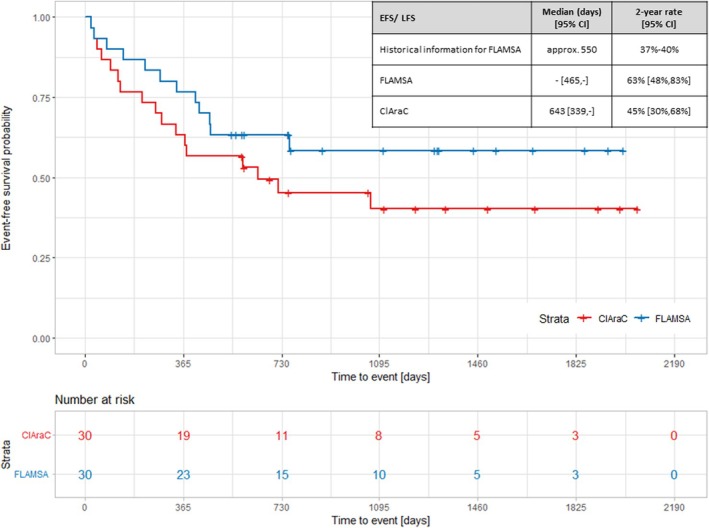
Results for event‐free survival of the ClAraC *vs.* FLAMSA treatment group. Results of Kaplan–Meier analysis for the event‐free survival (EFS) of the ClAraC and the FLAMSA treatment group. Displayed are median survival and 2‐year event rates with 95% confidence intervals (CI) for EFS/LFS (leukaemia‐free survival) for ClAraC, FLAMSA as well as information on historical controls.

The complete result for the primary endpoint is shown in Figure 2. Even without a statistical assessment, it is immediately obvious that the study failed to demonstrate superiority of the ClAraC treatment. In fact, a clear benefit was observed for patients treated with the current standard of care, FLAMSA. The results and conclusions for further endpoints and OS consistently favoured the FLAMSA treatment regimen (see Appendix, Figure S1). The standard treatment regimen leads to better outcome compared to what had been achieved in previous trials for the same standard. Reasons beyond patient selection are speculative, but improvements in co‐medication and diagnostics may be part of the explanation for this large difference when investigating the same treatment in the same disease setting.

## 3 DISCUSSION

This case study in haematology highlights that results from a RCT can be informative, even if the trial is formally underpowered. The results of the RCT provide direct information for the comparison of the experimental treatment with the control. In contrast, the results of a SAT can only be put into perspective to the planning assumptions or the data underlying these assumptions. At the end, our research question is directly answered with one randomized trial, because of the direct availability of the FLAMSA control group for assessment.

Despite being reliable, informative and fostering new developments in medicine, RCTs have never been particularly popular. Many reasons may exist, but probably the contrast between a human experiment with tossing a coin to decide about treatment on one side, and all training of physicians towards optimized individual decision‐making about which treatment benefits a specific patient best are extremes, which are difficult to overcome, even in the interest of science and the scientific treatment of a patient population. Other reasons may include trial logistics, costs and organizational aspects that are perceived as too burdensome. Oncology has a long‐standing tradition for the conduct of RCTs for providing confirmatory evidence of efficacy and a positive benefit/risk. We, like others, have observed this shift from accepting the randomized study as the gold standard towards (at an extreme) asking for a justification for including a patient in a RCT or trying to compensate the lack of direct control in a trial with a better molecular/mechanistic understanding of the drug's efficacy. This is concerning, particularly in indications where prognosis of patients is often poor, and it may be particularly difficult to decide, whether a patient died despite or because of treatment based on information from a SAT or when treating future patients outside a controlled trial.

We reported a case study where initially a SAT had been planned, but then, after a careful scientific discussion, more arguments spoke in favour of controlling the trial. The planning assumptions regarding the current standard were well founded in literature and were approximately met by the experimental combination. As such, the trial had formally failed its primary objective. However, positive findings regarding tolerability, combined with the remaining ambiguity about the outcome of SATs (this time speaking for continuing research), may have led to the initiation of a RCT. We argued earlier that this may be a waste of resources and a risk to study patients, but we lacked a concrete example, which we present now with the current study.^14^ Different standards for earlier phases of drug development and small populations should not be acceptable,^15^ because with limited sample size in practice, the likelihood of the second trial being conducted after a SAT is available is even lower. This may go both directions: Interest may calm down after results from a SAT are available, even though an experimental drug may have brought forward the field in the concept of a small step in the right direction. Or, interest (or the perceived need to directly change the treatment recommendation) may be felt too high to engage in another (likely even longer) trial.

It is a misconception that a SAT ‘maximizes’ the information provided by the available patients. In addition, the conduct of a SAT grossly neglects avoidance of bias and lacks comparative standards. In consequence, a randomized design should be used whenever feasible and is only obsolete when the expected treatment effect is enormous.^16^ Notably, dramatic effects and miracles are rare, and in consequence, to answer most research questions reliably, a RCT would be the best option, especially when the number of patients available is small.

## 4 CONCLUSION

The current proposed revision of the European Union's pharmaceutical legislation states that clinical trials ‘shall be done as “controlled clinical trials” if possible, randomised […]’.^17^ A detailed justification for the conduct of a SAT is often not provided. The reliability and risks to a straightforward interpretation of trial outcomes should be thoroughly discussed at the planning stage not only from the perspective of the intended/hoped for trial outcome. Instead, potential risks should be acknowledged, as well. In particular, in situations with high‐unmet medical need the hurdle of conducting a RCT is even higher after ‘promising results’ from a SAT are available. Chalmers' mandate to randomize the first patient is extreme,^18^ but high investments in large SAT are unwarranted given that small RCTs can be informative.

## AUTHOR CONTRIBUTIONS

GB and AGa were principal investigators of the clinical trial and TF, AGr, and AK were the trial statisticians. AK conceived the idea for this paper. TF and AGr drafted the manuscript. All authors contributed to the manuscript, critically revised it and consented to the final version of the manuscript.

## CONFLICT OF INTEREST STATEMENT

All authors have no commercial or financial conflicts of interest.

## Supporting information


**Table S1:** Baseline characteristics of the ClAraC‐SCT study.
**Table S2:** Main results of safety analyses of the ClAraC‐SCT study.
**Figure S1:** Results for overall survival of the ClAraC vs FLAMSA treatment group.

## Data Availability

The data that support the findings of this paper are available from the Department for Hematology, Hemostasis, Oncology and Stem Cell Transplantation of the Hannover Medical School upon reasonable request.
