# Supplementary material for: Randomization in phase II trials: No exemption based on sample size
Source: Br J Clin Pharmacol. 2025 Jul 8;91(9):2750–4. doi: 10.1002/bcp.70167 (PMC12381617; doi:10.1002/bcp.70167)

**Supplementary Appendix**

This document includes Supplementary Material to:

# Randomization in phase II trials: No exemption based on sample size

Theodor Framke, Gernot Beutel, Arnold Ganser, Armin Koch, Anika Großhennig

**Table of Contents**

Supplementary Table 1: Baseline characteristics. ........ 3
Supplementary Table 2: Main results of safety analyses. ........ 4
Supplementary Figure 1. Results for overall survival of the ClAraC vs FLAMSA treatment group. ........ 5

**Supplementary Table 1: Baseline characteristics of the ClAraC-SCT study.**

| | ClAraC (n=30) | FLAMSA (n=30) | Total (n=60) |
|---|---|---|---|
| Sex (female) | 16 (53.3%) | 17 (56.7%) | 33 (55.0%) |
| Race | | | |
| Caucasian | 30(100%) | 28 (93.3%) | 58 (96.7%) |
| Asian | 0 (0%) | 1 (3.3%) | 1 (1.7%) |
| Other | 0 (0%) | 1 (3.3%) | 1 (1.7%) |
| Remission stratum: AML with complete remission | 9 (30.0%) | 8 (26.7%) | 17 (28.3%) |
| Study site | | | |
| Site 1 | 21 (70.0%) | 21 (70.0%) | 42 (70.0%) |
| Site 2 | 6 (20.0%) | 7 (23.3%) | 13 (21.7%) |
| Site 3 | 3 (10.0%) | 2 (6.7%) | 5 (8.3%) |
| Height [cm] | 172.2±9.5 | 173.1±9.4 | 172.6±9.4 |
| Weight [kg] | 76.2±19.6 | 79.3±17.6 | 77.8±18.5 |
| Body Surface Area | 1.89±0.28 | 1.95±0.24 | 1.92±0.26 |
| Age [years] | 55.1±9.9 | 53.0±10.4 | 54.1±10.1 |
| HLA-match (HLA-identical) | 24 (80.0%) | 29 (96.7%) | 53 (88.3%) |
| Donor (related) | 8 (26.7%) | 7 (23.3%) | 15 (25.0%) |
| ECOG | | | |
| Grade 0 | 15 (53.6%) | 17 (58.6%) | 32 (56.1%) |
| Grade 1 | 10 (35.7%) | 8 (27.6%) | 18 (31.6%) |
| Grade 2 | 3 (10.7%) | 4 (13.8%) | 7 (12.3%) |

**Supplementary Table 2: Main results of safety analyses of the ClAraC-SCT study.**

| | ClAraC | FLAMSA |
|---|---|---|
| Cardiac toxicity | 26 / 30 (86.7%) | 27 / 30 (90.0%) |
| Rate of engraftment | 28 / 30 (93.3%) | 28 / 30 (93.3%) |
| Total number of AEs | 874 | 711 |
| … number of AE with Outcome: not recovered (persistently not changing) | 22 / 874 (2.5%) | 8 / 711 (1.1%) |
| … number of AE with suspected causal relationship to study medication | 434 / 874 (49.7%) | 366 / 711 (51.4%) |
| Number of patients with at least one | | |
| … AE | 30 / 30 (100%) | 30 / 30 (100%) |
| … AE with Outcome: fatal | 3 / 30 (10.0%) | 2 / 30 (6.7%) |
| … AE with Outcome: not recovered (persistently not changing) | 5 / 30 (16.7%) | 1 / 30 (3.3 %) |
| … AE with suspected causal relationship to study medication | 29 / 30 (96.7%) | 20 / 30 (96.7%) |
| Total number of SAEs | 20 | 18 |
| Number of patients with at least one SAE | 7 / 30 (23.3%) | 7 / 30 (23.3%) |

**Supplementary Figure 1. Results for overall survival of the ClAraC vs FLAMSA treatment group.** Results of Kaplan-Meier analysis for the overall survival (OS) of the ClAraC and the FLAMSA treatment group. Displayed are median survival and 2-year event rates with 95% CI (confidence intervals) for OS for ClAraC, FLAMSA and information of historical control.

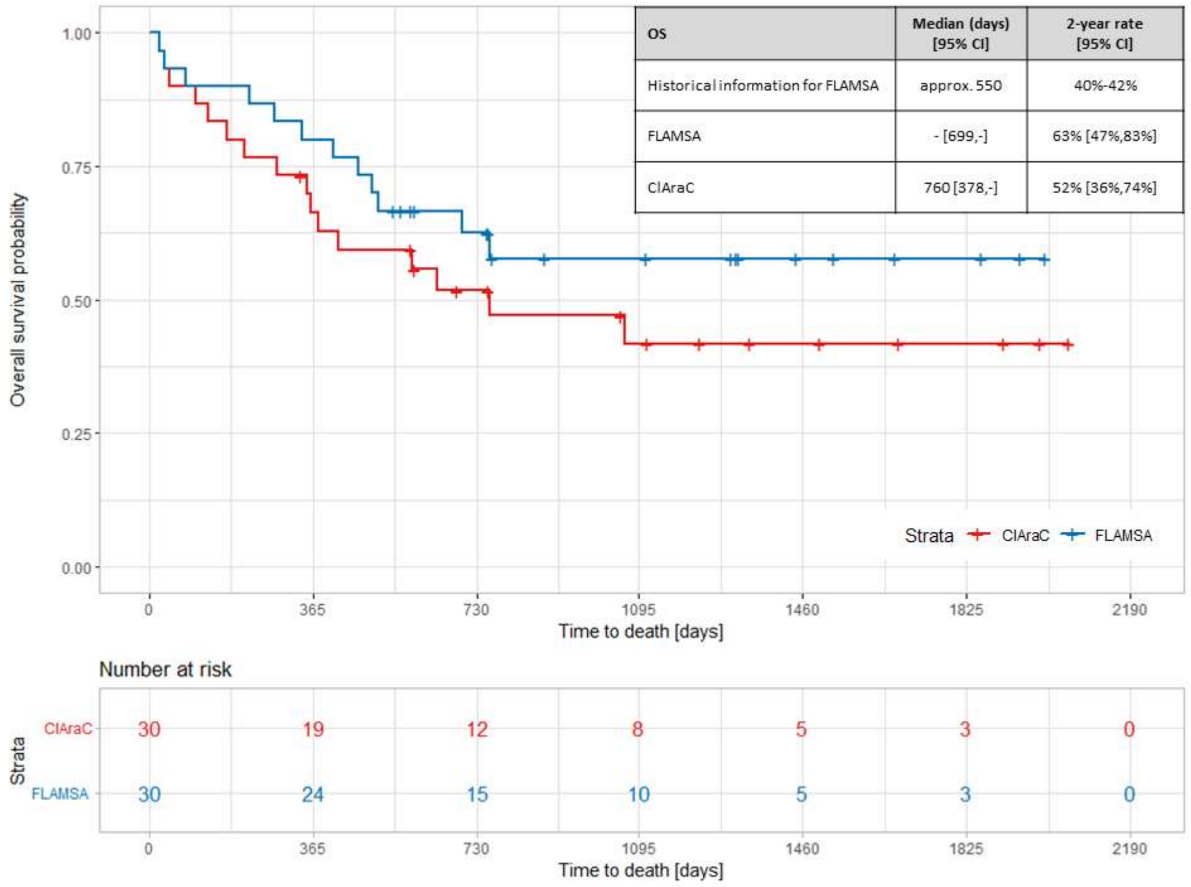

Supplement: Supplementary file 1 — Table S1: Baseline characteristics of the ClAraC‐SCT study. Table S2: Main results of safety analyses of the ClAraC‐SCT study. Figure S1: Results for overall survival of the ClAraC vs FLAMSA treatment group. [file BCP-91-2750-s001.docx]
